# The first metatarsophalangeal joint meniscus

**DOI:** 10.1186/1757-1146-4-S1-P53

**Published:** 2011-05-20

**Authors:** Dean J Samaras, Andrew C Kingsford

**Affiliations:** 1 Kingsford Podiatry Group, Melbourne, Victoria, 3000, Australia

## 

An intra-articular ligament of the first metatarsophalangeal joint (MTPJ) has been observed by the authors during dissection for hallux valgus and hallux rigidus corrective surgery over a number of years. The enigmatic incidence and role of this structure prompted further investigation. A literature review was conducted across several scientific databases using the search terms “first metatarsophalangeal joint” AND “meniscus” OR “anatomy” OR “plica” OR “intra-atricular ligament” OR “ligament”. In an effort to procure additional information several anatomy textbooks including those specific to the foot and ankle were obtained by the primary author for inspection, as were the expert opinions of several fellows of the Australasian College of Podiatric Surgeons. The literature search yielded just two papers pertaining to this structure and no reference had been made in any of the reviewed anatomy texts. One paper made reference to the structure in a case study and proposed it may be an additional ligament or meniscoid tissue. The authors of the second paper found a clear anatomical distinction (origin and insertion) and named it the meniscus of the first MTPJ as it showed histological resemblance to the meniscus in the knee. They proposed the transverse nature of the meniscus may assist in the stability of the joint preventing progression of hallux valgus deformity. On the contrary, the highest incidence of this structure was observed in feet with hallux valgus deformity. No mention has been made of the incidence or role of the meniscus in hallux rigidus pathology. Further research is therefore required to understand the incidence, function and role of the meniscus in the pathology of the first MTPJ.

